# A comparison of machine learning methods for survival analysis of high-dimensional clinical data for dementia prediction

**DOI:** 10.1038/s41598-020-77220-w

**Published:** 2020-11-23

**Authors:** Annette Spooner, Emily Chen, Arcot Sowmya, Perminder Sachdev, Nicole A. Kochan, Julian Trollor, Henry Brodaty

**Affiliations:** 1 School of Computer Science and Engineering, UNSW Sydney, Sydney, Australia; 2 School of Psychiatry, UNSW Sydney, Sydney, Australia; 3 Centre for Healthy Brain Ageing (CHeBA), UNSW Sydney, Sydney, Australia; 4 Department of Developmental Disability Neuropsychiatry, School of Psychiatry, UNSW Sydney, Sydney, Australia

**Keywords:** Statistics, Dementia, Neurodegenerative diseases, Diseases, Data mining, Machine learning

## Abstract

Data collected from clinical trials and cohort studies, such as dementia studies, are often high-dimensional, censored, heterogeneous and contain missing information, presenting challenges to traditional statistical analysis. There is an urgent need for methods that can overcome these challenges to model this complex data. At present there is no cure for dementia and no treatment that can successfully change the course of the disease. Machine learning models that can predict the time until a patient develops dementia are important tools in helping understand dementia risks and can give more accurate results than traditional statistical methods when modelling high-dimensional, heterogeneous, clinical data. This work compares the performance and stability of ten machine learning algorithms, combined with eight feature selection methods, capable of performing survival analysis of high-dimensional, heterogeneous, clinical data. We developed models that predict survival to dementia using baseline data from two different studies. The Sydney Memory and Ageing Study (MAS) is a longitudinal cohort study of 1037 participants, aged 70–90 years, that aims to determine the effects of ageing on cognition. The Alzheimer's Disease Neuroimaging Initiative (ADNI) is a longitudinal study aimed at identifying biomarkers for the early detection and tracking of Alzheimer's disease. Using the concordance index as a measure of performance, our models achieve maximum performance values of 0.82 for MAS and 0.93 For ADNI.

## Introduction

Data collected from clinical trials and cohort studies, such as dementia studies, are often high-dimensional, censored, heterogeneous and contain missing information, presenting challenges to traditional statistical methods. There is an urgent need for methods that can overcome these challenges to model this complex data.

The number of people living with dementia worldwide is now around 50 million and that number is forecast to triple by 2050 in the absence of any medical breakthrough. Researchers do not yet have a clear understanding of the risk factors for dementia despite decades of intensive study. There is currently no cure for dementia and no treatment that can successfully change its course. Machine learning models that can predict the time until a patient develops dementia are important tools in helping understand dementia risks and can give more accurate results than traditional statistical methods when modelling high-dimensional, heterogeneous, clinical data.

Survival analysis is a statistical method that aims to predict the time to an event, such as death, the diagnosis of a disease or the failure of a mechanical part. A key aspect of survival analysis is the presence of censored data, indicating that the event of interest has not occurred during the study period. The presence of censored data requires the use of specialised techniques. Traditionally, the Cox proportional hazards model^1^ has been the most widely used technique for analysing censored data, but the Cox model was designed for small data sets and does not scale well to high dimensions. Machine learning techniques that inherently handle high-dimensional data have been adapted to handle censored data, allowing machine learning to offer more flexible alternatives for analysing high-dimensional, right-censored, heterogeneous data.

Data is defined as high-dimensional when the number of features or variables exceeds the number of observations. In this case some methods of analysis become infeasible as the number of coefficients to be estimated exceeds the number of observations from which to estimate them, and so a unique solution cannot be found^2^. A general rule of thumb in survival analysis is that the number of events per variable (EPV) should be at least 10. For rare or infrequent events, it can be difficult to gather sufficient data to meet this requirement. Any dataset where this requirement is not met can be considered high-dimensional. Breakthroughs in medical technology mean that high-dimensional data is becoming more and more prevalent. High-dimensional data presents challenges to analysis including the strong risk of overfitting the model to the data, limiting the model's ability to generalise to new data, and the high variance of the models fitted to this data^3^.

Clinical data, collected from clinical trials and cohort studies that focus on the prevention, detection, treatment or management of various diseases and medical conditions, are typically not only high-dimensional and censored, but also heterogeneous in nature, with data arising from a variety of different sources with varying statistical properties, and containing missing information. Recent research has shown that different sources of clinical data can provide complementary information about dementia and that the integration of multiple sources of data leads to better prediction of cognitive decline than the use of a single source^4^. There is an urgent need for methods that can overcome the challenges presented by high-dimensional, clinical data.

The aim of this work is to systematically compare the performance and stability of a selection of machine learning algorithms and feature selection methods that are suitable for high-dimensional, heterogeneous, censored, clinical data, in the context of cognitive ageing and dementia, by predicting survival time to dementia.

The literature abounds in studies using machine learning for the automatic classification of Alzheimer's disease^4^, one form of dementia, with high levels of accuracy achieved. But dementia develops over a significant period of time, possibly decades^5^, so a study participant who does not develop dementia during the study period may not necessarily be free of the disease and that participant's data are considered censored. Therefore, survival analysis is a more appropriate method of modelling this data, where the time to the diagnosis of dementia may be unknown.

Few authors have systematically compared methods for high-dimensional survival analysis. Early work focussed on homogeneous gene expression data using mainly statistical modelling techniques. Bøvelstad et al.^6^ compared univariate feature selection, forward stepwise selection, both standard and supervised principal components regression, partial least squares regression and ridge and lasso regression on three different high-dimensional genomic data sets. Witten et al.^7^ examined a similar selection of methods but added clustering and additional variance-based methods. Van Wieringen et al.^8^ compared univariate Cox, supervised principal component analysis, penalised least squares, penalised Cox regression and tree-based ensemble methods, again on three different high-dimensional genomic data sets.

More recently Leger et al.^9^ examined a wide selection of machine learning algorithms and feature selection methods for survival analysis, on numeric data extracted from computed tomography (CT) scans of patients with head and neck squamous cell carcinoma. The authors included both parametric and non-parametric survival models. However, some of the methods used, such as the Pearson and Spearman's rank correlation coefficients, are not appropriate when applied to censored data^10^.

Recent works have begun to compare the performance of survival models on heterogeneous data but have mainly used Electronic Health Records (EHR), where the number of observations is generally far larger than in cohort studies. Steele et al.^11^ compared statistical modelling and machine learning approaches in EHR, using Cox regression models, random survival forests and elastic net regression. De Bin et al.^12^ examined methods to combine low-dimensional clinical and high-dimensional omics data using univariate feature selection, forward stepwise selection, the LASSO, and boosting. Posterl et al.^13^ investigated survival analysis of heterogeneous, high-dimensional medical data, but focussed on feature extraction, rather than feature selection.

Our work analyses data from both the Sydney Memory and Ageing Study (MAS)^14^, and the Alzheimer's Disease Neuroimaging Initiative (ADNI)^15^. MAS is a longitudinal cohort study, one of the largest studies of its kind, aimed at determining the effects of ageing on cognition. ADNI is a longitudinal study designed to identify biomarkers for the early detection of Alzheimer's disease (AD). Both data sets contain an extensive and diverse collection of heterogeneous data.

Only a few authors have applied machine learning techniques to data from MAS. Cui et al.^16,17^ predicted the development of mild cognitive impairment (MCI), a prodromal phase of dementia, using support vector machines applied to neuropsychological test scores and neuroimaging measures. Senanayake et al.^18,19^ compared a selection of machine learning algorithms, including support vector machines, random forests, Adaboost and ensemble methods, to discriminate between the various MCI subtypes. Neither of these authors used survival analysis, but instead treated the problem as a classification problem.

Other authors have applied survival analysis to the MAS dataset, using traditional statistical techniques rather than machine learning. They have analysed a small subset of the available data, so have not had to deal with the problems of high-dimensional data. Kochan et al.^20^ used Cox proportional hazards models to investigate intra-individual variability in cognitive performance as a predictor of mortality in old age. Connors et al.^21^ used the Cox proportional hazards model to determine whether a decline in cognitive ability predicts mortality in older individuals without dementia. Heffernan et al.^22^ used Cox regression to examine whether alcohol consumption is associated with incident dementia and whether APOE ε4 status plays a role in this relationship.

The application of machine learning techniques for survival analysis to data from the ADNI is also relatively limited. Orozco-Sanchez et al.^23^ explored several machine learning strategies for building Cox models, including penalised Cox regression, a univariate filter on the Cox Model and two model selection strategies. Li et al.^24^ developed a multi-task learning-based survival analysis framework that handles block-wise missing data. Several authors^25–27^ have developed neural networks for survival analysis on data from the ADNI. However, a comprehensive survey paper on the topic of machine learning for survival analysis does not include any studies employing these techniques for the prediction of Alzheimer's disease^28^.

As far as we can ascertain, this work is the largest comparison of methods for high-dimensional, heterogeneous survival analysis to date, across the broadest array of heterogeneous data. It is the first work to apply these methods to data from MAS and examines the most diverse variety of data in a study on dementia to date.

## Results

### Performance of the machine learning and feature selection models

**Figure 1:**
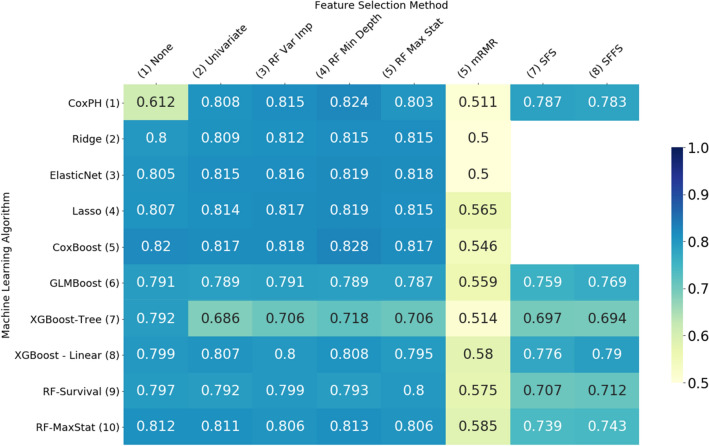
Heatmap illustrating the performance of each of the machine learning algorithms with each feature selection method on the MAS dataset, measured by the mean concordance index.

The results of our experiments are given in Fig. 1 (MAS) and 2 (ADNI), in the form of heatmaps that show the mean value of the concordance index (C-Index) across 5 repeats of 5-fold cross-validation for each combination of machine learning algorithm (rows) and feature selection method (columns). More details of these results are included in Supplementary Table S6 online. The machine learning algorithms can be divided into 3 main groups –penalised Cox regression models (rows 2–4), boosted survival models (rows 5–8) and random survival forests (rows 9–10). The Cox proportional hazards model (row 1), while not a machine learning algorithm, is included here as a benchmark against which to compare the other models.

Predictive performance was higher on the ADNI data than on the MAS data. In general, with the exception of the mRMR filter, all algorithms evaluated performed well on both data sets and outperformed the Cox proportional hazards model. There was not a single model which clearly outperformed all others, but this indicates that there are a range of alternative methods that can be used to analyse high-dimensional survival data.

**Figure 2:**
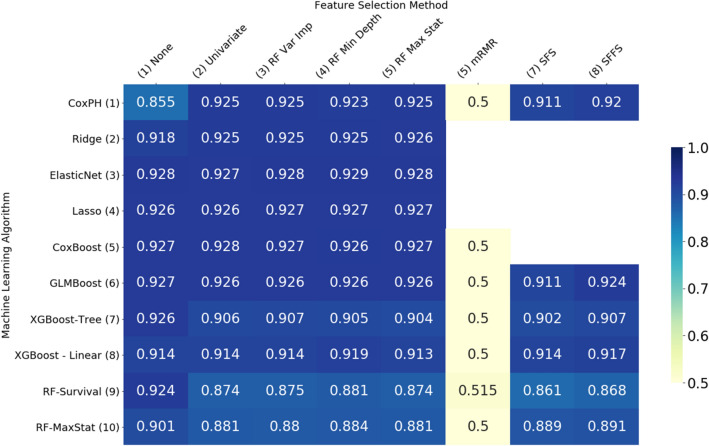
Heatmap illustrating the performance of each of the machine learning algorithms with each feature selection method on the ADNI dataset, measured by the mean concordance index.

Results were similar across the two data sets. In both cases the worst performing model was the Cox proportional hazards model (CoxPH—row 1, column 1). Without external feature selection, in the case of MAS the best performing learner was the Cox model with likelihood-based boosting (Fig. 1, row 5, column 1) and for ADNI it was the ElasticNet (Fig. 2, row 3 column 1).

There was little to differentiate the penalised regression models (rows 2–4) in either data set, especially when feature selection was applied. In both cases the Ridge was the weakest performer, but only by a small margin, despite the fact that several authors^6,8^ have found that Ridge regression outperforms LASSO in the high-dimensional setting.

Of the boosted models (rows 5–8), the strongest performer was the Cox model with likelihood-based boosting (CoxBoost, row 5), although in the ADNI data set, the results on the Cox model with gradient boosting (GLMBoost, row 6) were almost identical. Extreme gradient boosting with linear model-based boosting (XGBoost—Linear, row 8) outperformed the same method with tree-based boosting (XGB—Tree, row 7) in almost all cases. In the MAS data set, extreme gradient boosting with tree-based boosting did not perform well when feature selection methods were applied.

The maximally selected rank statistics random forest (RF-MaxStat, row 10) outperformed the random survival forest (RF—Survival, row 9) in almost all cases.

Of the feature selection methods tested (columns 2 to 9), the random forest minimum depth filter (RF Min Depth, column 4) produced the most accurate models, although there was little difference in performance between any of the random-forest-based feature selection methods. The mRMR filter (mRMR, column 6) produced the least accurate results.

Tests of statistical significance were applied in a pairwise fashion to the results. While the performance of the evaluated models was generally high, there was not a statistically significant difference in performance between many of the best-performing models. Supplementary Table S6 online shows those models whose performance was significantly worse than the best-performing model.

### Stability of the models

Stability of a machine learning algorithm is defined as the sensitivity of a method to variations in the training set^29^. An algorithm is considered unstable if a small change in the training set causes a large change in the performance of the algorithm. The more stable an algorithm, the more reliable are its results and the greater the confidence in the results. It is not sufficient for an algorithm to perform well on a test dataset, ideally it must also be stable.

The boxplots in Supplementary Figures S7 and S8 online show the spread of values of the C-index achieved by each of the models over 5 repeats of 5-fold cross-validation. The centre line represents the median value, the upper and lower limits of the box represent the upper and lower quartiles and the whiskers represent the highest and lowest values.

One measure of stability is the standard deviation of the results from the 5 repeats of 5-fold cross-validation. These values are shown in brackets in Supplementary Table S6 online. For the ADNI data set, the standard deviation of the results is generally in the range 0.01 to 0.02, which is quite low. Many models achieved the minimum standard deviation of 0.01. For the MAS data set, on the other hand, the minimum standard deviation of 0.04 was achieved by sequential forward selection on the Cox model with gradient boosting. Most other models achieved a standard deviation of 0.05 to 0.06.

### Most predictive features

Using 5 repeats of 5-fold cross-validation, each model is run 25 times, so each feature could be selected by a model up to 25 times. For sparse models, i.e. those that select a subset of relevant features, rather than ranking all features, we recorded the total number of times each feature was selected by a model and ranked the features by that number. Intuitively, the more often a feature is selected by a model, the more predictive it must be.

The features that were selected most consistently by the sparse models in both data sets were the results of the neuropsychological test scores. Of the top 15 selected features, 11 in the MAS data set and 9 in the ADNI data set were neuropsychological test scores. In particular, results from the Logical Memory Test, the Verbal Fluency Test, Rey's Auditory Verbal Learning Test, the Digit Symbol Test and the Trail Making Test Part B all appeared in the top 15 selected features in both data sets. These objective scores ranked more highly than any of the subjective scores, which are obtained via self-assessment tools such as questionnaires.

The remainder of the top 15 selected features in the MAS data set included screening tools such as the Informant Questionnaire on Cognitive Decline in the Elderly *(*IQCODE) and the General Practitioner assessment of Cognition (GPCOG), participant age and the results of the Brief Smell Indicator Test (BSIT).

In the ADNI data set, the remaining top 15 selected features included the Clinical Dementia rating (global and sum of boxes scores), the Mini Mental State Exam (MMSE) score (top 20 in MAS), the year of onset of AD symptoms, whether the patient was on medication for dementia and the Functional Assessment Questionnaire. These selections are consistent with the Alzheimer's disease literature^14,30^.

A more detailed analysis of the features selected by the models is reserved for a future publication as this is an important subject that merits more discussion than can be included here.

## Discussion

With advances in modern data collection techniques producing ever larger clinical data sets, it is essential to identify methods that can be used to analyse high-dimensional, heterogeneous, survival data. We therefore evaluated a range of machine learning algorithms capable of analysing this type of data.

Performance on the ADNI data set was overall higher than on the MAS data set. This was to be expected as the ADNI is a clinical trial where the characteristics of the participants can be strictly controlled. MAS, on the other hand, is a population-based study and participants are likely to have a range of co-morbidities. As such, results from MAS should be more generally applicable to the population as a whole.

In general, our results show that machine learning can provide more accurate alternatives to traditional methods for survival analysis, such as the Cox proportional hazards model, in the presence of high-dimensional data. The Cox model is not designed to handle high-dimensional data and the large number of features in the data sets analysed causes the model to overfit the data, thus reducing the accuracy of the results for unseen data. However, applying feature selection techniques before using the Cox proportional hazards model improved its performance to a level that was comparable with the machine learning algorithms that use embedded feature selection.

While the performance of the evaluated models was generally high, there was not a statistically significant difference in performance between many of the best-performing models, indicating that there are many powerful algorithms to choose from. In this case, we can look at other characteristics of the models to help make a selection.

The Cox model with likelihood-based boosting is one of the best predictors in both data sets, either on its own or with feature selection. A significant advantage of this model is its ease of use. The algorithm automatically calculates the number of boosting steps to perform and in doing so is resistant to overfitting. This is likely to be one of the reasons for its high performance, but also means that the user does not need to tune any hyperparameters, making it easy to achieve a high-performing model. In contrast, the random forests and extreme gradient boosting offer many hyper-parameters that can be finely tuned, but which make model development more difficult.

The Random Forest Minimal Depth feature selector is another method that performs well on both data sets. The Minimal Depth algorithm measures the predictiveness of variables and automatically sets a cut-off threshold that is used to separate the strong (non-noisy) variables—those that affect the survival outcome—from those that are not relevant. Its inbuilt ability to calculate this threshold and the fact that it inherently handles high-dimensional data are the main reasons for its success.

In many cases, feature selection did not improve the performance of the base model. This is likely to be because we have chosen methods that are already suited to handling high-dimensional data, and so do not require initial feature selection. Intuitively, many of these models perform their own internal feature selection while building the model, so perhaps they benefit from having access to the entire set of features during model building.

**Figure 3:**
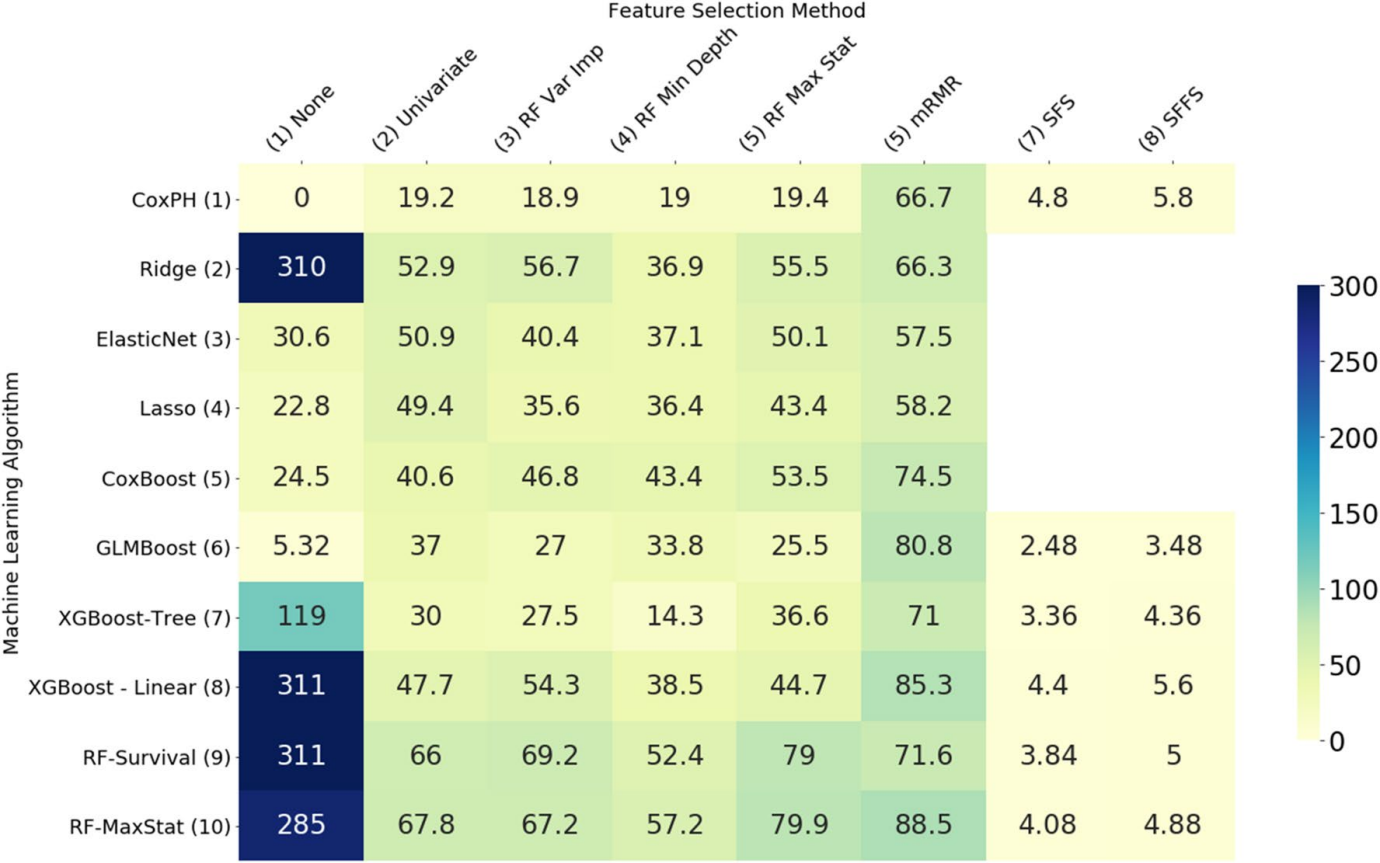
Average number of features selected by each model—MAS dataset.

**Figure 4:**
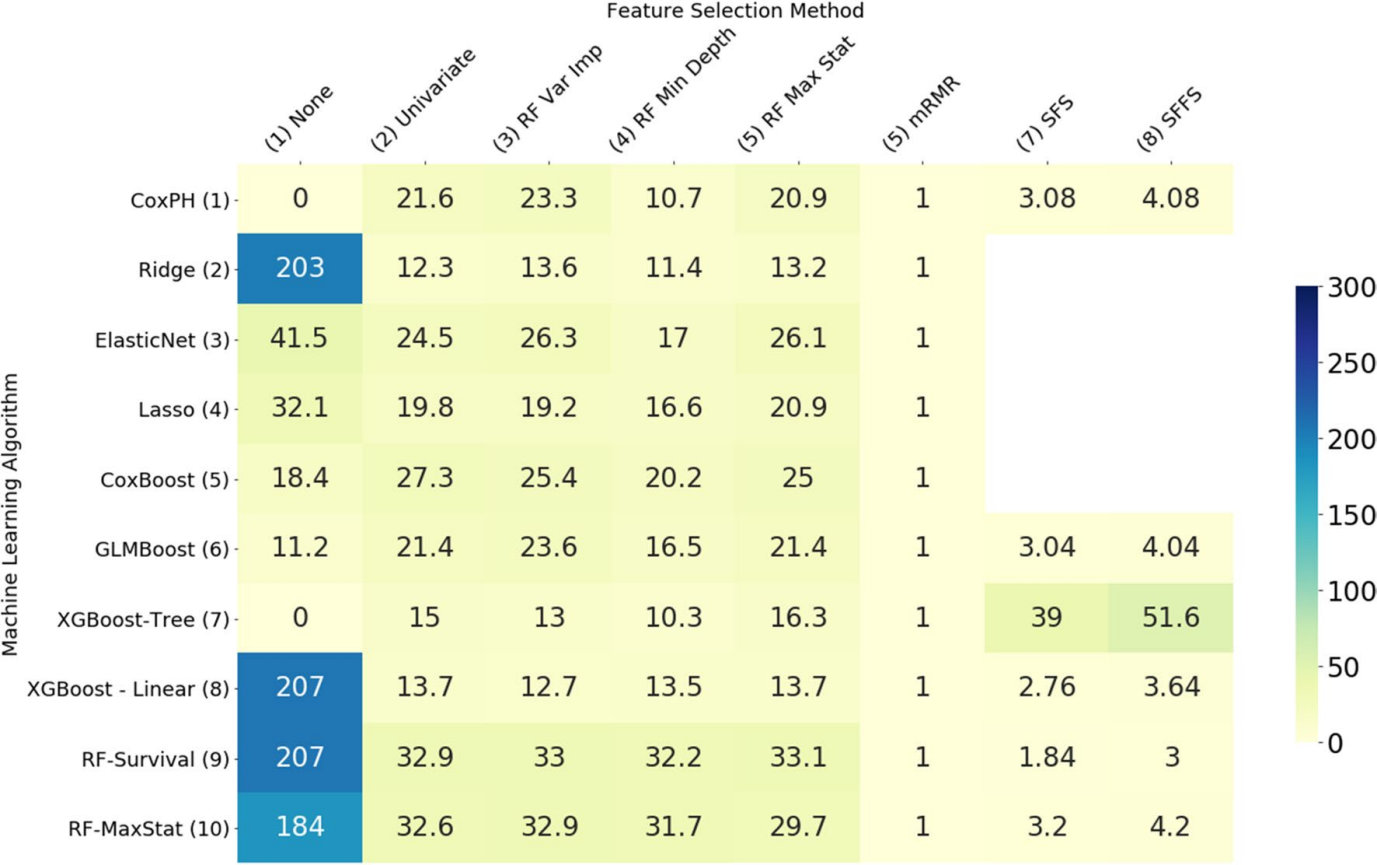
Average number of features selected by each model—ADNI dataset.

The variation in performance of the feature selection methods evaluated can be explained, at least in part, by the number of features selected by each method, shown in Figs. 3 and 4. In general, the most accurate models were those which selected a moderate number of features—in the mid range of about 10–30 on the ADNI data set and about 40–60 on the MAS data set. Where algorithms selected more features than these mid ranges, the additional features are not only likely to be less relevant but also cause the model to overfit the data, giving poorer results when generalising to new data. Where fewer features were selected, some of the more important features were likely omitted.

Both Random Forests and boosted models can detect complex, non-linear relationships amongst the features, giving them an advantage over linear regression models when non-linear data is present. But in this case, not only do the linear regression models outperform the random forests, but the extreme gradient boosting models using a linear booster outperforms that using a tree-based booster in most cases as well. This could indicate a linear relationship amongst the features.

Based on the results achieved here, the Cox model with likelihood-based boosting (CoxBoost) is the preferred machine learning algorithm to analyse high-dimensional, censored data, and the Random Forest Minimal Depth algorithm is the best-performing method for feature selection.

The work described here focuses on the performance of machine learning and feature selection algorithms for high-dimensional, censored, clinical data. Another area that needs further research is the selection of a suitable cut-off threshold for the feature selection algorithms, to ensure that only relevant features are included in the final model.

## Methods

### Participant cohorts

#### Sydney Memory and Ageing Study (MAS)

Subjects were selected from the Sydney Memory and Ageing Study (MAS), a longitudinal cohort study aimed at examining the characteristics and prevalence of mild cognitive impairment and dementia. MAS was initiated in 2005, randomly recruiting 1037 community-dwelling adults, aged 70–90 years at baseline, from the electoral roll of two federal government electoral areas in eastern Sydney, Australia. Full details of the study can be found^14^.

Ethics approval for the study was granted by the Human Research Ethics Committees of the University of New South Wales and the South Eastern Sydney and Illawarra Area Health Service. All participants and informants gave written informed consent. The MAS study and this work were carried out in accordance with the MAS Governance guidelines, which are based on relevant University of New South Wales and National Health and Medical Research Council research and ethics policies.

Cognitively normal classifications were only applied to participants from English-speaking backgrounds since the available normative data are based on predominantly English speakers. As a result, only participants from an English-speaking background were used in the models. This reduced the cohort size to 837.

### Alzheimer's Disease Neuroimaging Initiative (ADNI)

The ADNI was launched in 2003 as a public–private partnership, led by Principal Investigator Michael W. Weiner, MD. The primary goal of ADNI has been to test whether serial magnetic resonance imaging (MRI), positron emission tomography (PET), other biological markers, and clinical and neuropsychological assessment can be combined to measure the progression of mild cognitive impairment (MCI) and early Alzheimer’s disease (AD).

Subjects who participated in ADNI phase 1 were selected for this study.

The characteristics of both studies are summarised in Table 1.

**Table 1:** Study characteristics.

	MAS	ADNI-1
Study design	Longitudinal, cohort study	Longitudinal, observational clinical trial
Sample size (n)	873	800
Number of features (p)	251	185
Censoring rate (%)	82	47
Follow-up period	2 years	6 months
Age at baseline	70–90 years	55–90 years

### Data

The MAS data set contains a diverse collection of data including demographics, status of the APOE gene, genetic risk scores, objective cognitive data including comprehensive neuropsychological test scores, subjective cognitive ratings, medical history, family history, medical examination, blood test results, psychological scores, functional data (including activities of daily living, physical, mental and social activity), nutritional data, quality of life ratings, and medications. We used only baseline MAS data.

The ADNI data set also contains a heterogeneous collection of data including demographics, status of the APOE gene, neuropsychological test scores, medical history, family history, medical examination, blood test results and adverse events. We used only data from ADNI phase 1 and selected data that most closely matched the data in MAS.

The use of baseline data in this study indicates that the survival analysis considered the time from the start of the study to the time of diagnosis of dementia or censoring.

### Data pre-processing

Data pre-processing was undertaken to prepare the data appropriately for the machine learning algorithms.

Clinical datasets typically contain missing values and the process of substituting appropriate values for the missing ones is known as imputation. A widely accepted method of imputing missing data is multiple imputation, which preserves the relations in the data and the uncertainty in those relations^31^. It is assumed throughout this work that the data are missing at random and that censoring is non-informative i.e. does not depend on the diagnosis of dementia. Missing data was imputed using the method of multiple imputation by chained equations in the R package *mice*^32^. Imputation was performed within the cross-validation loop, first on the training set and then on the test set using the prediction matrix developed on the training set.

The standard method of dealing with categorical data in machine learning is to use one-hot encoding, whereby a new binary feature is created for each level of each categorical feature. With a large number of categorical features, such as in the MAS dataset, this can increase the dimensionality of the data enormously. Moreover, categories with levels containing only a small number of observations are usually not very predictive.

In order to overcome these difficulties, alternative methods of dealing with categorical data were employed. Levels that contained only a small number of observations were combined, often creating a new binary feature indicating simply a normal or abnormal status for a medical marker, such as posture, instead of a categorical variable with multiple levels indicating varying degrees of severity of that marker. In addition, where several categorical features measured related conditions, such as a tremor in the head, face, right arm, left arm, at rest and active, the features and their levels were combined to create a single binary feature indicating the presence or absence of the condition e.g. the presence or absence of any tremor. One-hot encoding was used to transform any remaining categorical variables.

All continuous features were normalised. Where multiple readings were taken for the same measurement, e.g. pulse, those values were averaged. Any features that had more than 50% (MAS) or 55% (ADNI) of values missing were eliminated. Full details of these transformations can be found in Supplementary Tables S4 and S5 online.

Following data pre-processing the MAS dataset consisted of 251 features (311 after one-hot encoding), of which 21 were categorical, 103 boolean (or logical in R terminology) and the remainder numeric. The ADNI dataset consisted of 185 features (207 after one-hot encoding), of which 9 were categorical, 72 boolean and the remainder numeric.

### Model selection

Ten machine learning algorithms and eight feature selection methods capable of handling high-dimensional, heterogeneous, censored data were selected for evaluation. Each feature selection method was applied to each survival model where possible. The event of interest was a diagnosis of dementia at any time during the study period. The models were built at the population level and personalised models were not considered.

The machine learning algorithms selected can be divided into three categories:

1. Penalised Cox Regression: LASSO, ElasticNet and Ridge regression.
2. Boosted Cox Regression: Cox model with likelihood-based boosting (CoxBoost), Cox model with gradient boosting (GLMBoost) and Extreme Gradient Boosting (XGBoost) with tree-based and linear model-based boosting.
3. Random Forests: random survival forest, maximally selected rank statistics random survival forest.

A brief description of these algorithms can be found in Supplementary Methods S1 online.

The feature selection methods tested can similarly be divided into categories:

1. Filter Methods: univariate Cox score (Univariate), random forest variable importance (RF Var Imp), random forest minimal depth (RF Min Depth), random forest variable hunting (RF Var Hunt), maximally selected rank statistics random forest (RF Max Stat), minimum redundancy maximum relevance (mRMR).
2. Wrapper Methods: sequential forward selection (SFS), sequential forward floating selection (SFFS).

A brief description of these methods can be found in Supplementary Methods S2 online.

Many of the machine learning algorithms employed have one or more hyper-parameters that must be selected to optimise model performance. Tuning of these hyper-parameters was performed automatically within a 5-fold nested cross-validation loop. A random search with 25 iterations was used to select values for the hyper-parameters in the inner loop and model performance was evaluated in the outer loop. In this way all model selection steps were repeated for every pair of training/test data. A list of the hyper-parameters that were tuned can be found in Supplementary Table S3 online. This table also shows the R packages used to implement each method.

Where a filter was used to rank the features for the purpose of feature selection, a threshold to determine the number of features used in the final model was required. The threshold was determined by tuning within a 5-fold nested cross-validation loop, a random search with 25 iterations being used to select values for the threshold in the inner loop, while model performance was evaluated in the outer loop. A limitation of *mlr* is that when tuning a feature selector, it only allows tuning of the number of features to be selected, not both the number of features and the hyperparameters. To overcome this problem, we tuned the hyperparameters in the first round of experiments on the base models alone, and then used those hyperparameter values in the experiments on the feature selectors.

It was not possible to apply sequential forward selection to any of the penalised regression models as these models require at least two features and both sequential forward selection methods start with an empty model, adding in the first iteration only one feature at a time. When using the mRMR filter on the ADNI data set, only one feature was selected. So it was not possible to apply this filter to the penalised regression models for the same reason. It was also not possible to apply sequential forward selection to the boosted Cox regression algorithm as this became computationally infeasible.

The R package *mlr* (Machine Learning in R)^33^ was used as a framework to carry out benchmarking and all code for the experiments was written in R^34^. The visualisations were created in Python. All resampling was performed using 5 repeats of stratified 5-fold cross validation.

### Model evaluation

The metric used to evaluate the models was the concordance index (C-Index)^35^, which measures the proportion of pairs where the observation with the higher survival time has the higher probability of survival as predicted by the model.

When random sampling, such as cross-validation, is used, model performance can be sensitive to the particular random partitioning that results. For this reason, we have applied tests of statistical significance to the performance results of the models.

Generally, a paired t-test can be performed to test the null hypothesis that the mean of two sets of values is the same. However, when the two sets of values in question are the performance results of two models tested using random sampling, such as repeated k-fold cross-validation, the independence assumption of the t-test is violated, because the different training and test sets may overlap.

Dietterich^36^ examined five different statistical tests for comparing the results of supervised learning algorithms to determine their probability of Type I error. A Type I error is the probability of finding a significant difference when there is none—a false positive. A Type II error is the probability of not detecting a significant difference when there is one—a false negative. He recommended the use of the *5* × *2 cv test*, because of its acceptable Type I error and its higher power.

Nadeau and Bengio^37^ recognised that when performing a standard t-test on results from a repeated k-fold cross validation, the Type I error is inflated because the violation of the independence assumption leads to an underestimation in the variance. As a result, they proposed the *corrected resampled paired t-test*, which takes the lack of independence into account and corrects the variance.

When performing hypothesis tests with multiple comparisons, the *p*-values must be adjusted to prevent data from incorrectly appearing to be statistically significant. We have applied the *corrected resampled paired t-test* in a pairwise fashion to the results of our models, and adjusted the *p*-values using the R function p.adjust, with method “fdr” to control the false discovery rate^38^.

## Supplementary information

Supplementary information.

## Data Availability

Access to the MAS data can be requested by applying to the Sydney Memory and Ageing Study via memory@unsw.edu.au or chebadata@unsw.edu.au. Access to the ADNI data can be requested at https://adni.loni.usc.edu/data-samples/access-data/
