# Supplementary material for: A comparison of machine learning methods for survival analysis of high-dimensional clinical data for dementia prediction
Source: Sci Rep. 2020 Nov 23;10:20410. doi: 10.1038/s41598-020-77220-w (PMC7683682; doi:10.1038/s41598-020-77220-w)
Supplement: Supplementary file 1 — Supplementary information. [file 41598_2020_77220_MOESM1_ESM.docx]

**A Comparison of Machine Learning Methods for Survival Analysis of High-Dimensional Clinical Data for Dementia Prediction**

# Supplementary Material

Annette Spooner^1^*, Emily Chen^1^, Arcot Sowmya^1^, Perminder Sachdev^2,3^, Nicole A. Kochan^3^, Julian Trollor^2,3,4^, Henry Brodaty^2,3^ for the Alzheimer’s Disease Neuroimaging Initiative

^1^ School of Computer Science and Engineering, UNSW Sydney, Sydney, Australia.

^2^ School of Psychiatry, UNSW Sydney, Sydney, Australia.

^3^ Centre for Healthy Brain Ageing (CHeBA), UNSW Sydney, Sydney, Australia.

^4^ Department of Developmental Disability Neuropsychiatry, School of Psychiatry, UNSW Sydney, Sydney, Australia

### Supplementary Methods S1: Machine Learning Algorithms

Supervised machine learning algorithms do not inherently handle censored data as they rely on having an outcome in order to train a model to learn from that outcome. However, several machine learning algorithms have been adapted to work with censored data. A short description of these methods is provided below. The original articles may be consulted for more detailed information about these methods. The Cox Proportional Hazards Model, while not a machine learning algorithm, is included for comparison.

### Cox Proportional Hazards Model

The standard statistical tool for analysing censored survival data is the Cox proportional hazards model ^1^, which evaluates the effect of several variables simultaneously on the time to an event of interest, such as death or the diagnosis of a disease. While it is a robust model, the Cox model does not generalise well to high dimensions, applies only to linear data and is limited by some strict assumptions, such as the proportional hazards assumption, that may not hold true in the real world. It is included here as a baseline against which to compare the other models.

The Cox model is expressed by the hazard function, which is the risk of an event occurring at time t as follows:

*h*(*t*) = *h*_0_(*t*) × *exp*(*β*_1_*x*_1_+*β*_2_*x*_2_+...+*β_p_x_p_*)

where *t* represents the survival time, *h*(*t*) is the hazard function, {*x*_1_,*x*_2_,...,*x_p_*} are the values of the p covariates, {*β*_1_,*β*_2_,...,*β_p_*} are the coefficients that measure the effect of the covariates on the survival time and h_0_(t) is the baseline hazard function, which is unspecified.

The regression coefficients are estimated by maximising the partial likelihood.

### Penalized Cox Regression

Standard linear regression performs poorly when applied to high-dimensional data. To overcome this, a penalised model, that adds a constraint to the equation, is often used. This constraint reduces, or shrinks, the coefficient values towards zero, reducing their variance and ensuring that the less important features have less impact in the model.

The two most common forms of regularisation use an L_1_ or an L_2_ penalty constraint. An L_1_ penalty (also known as LASSO regression) is equal to the absolute value of the magnitude of coefficients. It can produce models with a smaller number of coefficients (sparse models) because some coefficients are reduced to zero and therefore eliminated. In this way the LASSO performs feature selection in addition to fitting the model. An L_2_ penalty (also known as ridge regression), is equal to the square of the magnitude of the coefficients. Ridge regression shrinks all coefficients by the same factor, and none are eliminated, so it does not produce sparse models.

The LASSO suffers from a number of limitations in that it cannot select more features than the number of samples and where there are correlated features, it tends to select only one from a group indiscriminately. ^2^ Elastic net regression ^2^, a linear combination of the L_1_ and L_2_ penalties of the LASSO and ridge methods, was developed to overcome these limitations and is known to be particularly useful when the number of features is larger than the number of samples.

LASSO, ridge and elastic net regression have all been extended to the Cox model ^3^ ^4^ and are evaluated here.

### Boosted Cox Regression

Boosting is an iterative technique that was developed in the machine learning community ^5^ and later adapted to statistical modelling, including survival analysis ^6^ ^7^. It is an ensemble technique that trains weak learners sequentially, where each new model that is added to the ensemble learns from the mistakes of the previous models. Boosting is resistant to overfitting, provided the number of boosting steps is carefully chosen, and it can cope with high-dimensional data.

There are two main approaches to boosting for statistical modelling, including survival analysis – likelihood-based boosting and gradient boosting. Likelihood-based boosting ^8^ uses base learners that maximise an overall likelihood in each boosting step, selecting only the base-learner which leads to the largest increase in the likelihood. Gradient boosting ^9^ is equivalent to iteratively re-fitting the residuals of the ensemble model at each step.

In this work, four different boosting algorithms are evaluated – CoxBoost, a likelihood-based boosting algorithm that uses the Cox model as its base learner, GLMBoost, a gradient boosting algorithm that uses penalised Cox regression models as its base learners, and XGBoost ^10^, or Extreme Gradient Boosting, an implementation of gradient boosting that is scalable and optimised for execution speed and model performance. XGBoost offers tree-based and linear model-based boosting and both methods are assessed.

### Random Forests

Random survival forests ^11^ are an extension of Breiman's random forest ^12^ to censored survival data. Random survival forests aggregate the results from many decision trees, each generated from a bootstrap sample of the data. At each node in the random forest, one feature is selected to split on, from a random subset of all features. In a random survival forest, the feature and split point chosen is the one that maximises the survival difference between daughter nodes i.e. that maximises the log rank statistic over all available split points and features.

Random survival forests are becoming more widely accepted as an alternative to the Cox proportional hazards model due to their ability to model complex, non-linear data, handle high-dimensional data, identify interactions and impute missing data naturally. As such they reduce the tendency for the model to overfit the data.

One drawback of the original random forest and the random survival forest, however, is that they are both biased toward selecting features with many possible split points to split on, e.g. categorical variables with several levels. The conditional inference forest ^13^, which separates the selection of the feature to split on from the selection of the split point, was introduced to overcome this problem. However, the split point selection in conditional inference forests is based on a linear rank statistic and so cannot identify non-linear effects in features considered for splitting.^14^ An alternative is to select the split point using maximally selected rank statistics.^15^ ^14^ Both the standard random survival forest and the maximally selected rank statistics random survival forest are evaluated.

## Supplementary Methods S2: Feature Selection Methods

Feature selection is the process of selecting a subset of relevant features. It is an essential part of analysing high-dimensional data and is used to enhance model performance, improve computational speed and enhance the interpretability of the model.

Feature selection techniques can be divided into filter, wrapper and embedded methods ^16^. Filter methods rank the features according to some external measure and then use a threshold to select the most important features. A model is then fitted to the subset of features that exceeded the threshold, to evaluate the performance of the filter. Wrapper methods, also known as feature subset selection, use the performance of a machine learning algorithm to evaluate the predictive power of subsets of features. Embedded methods perform feature selection as part of the model-building process. Many of the machine learning algorithms described in Supplementary Methods S1 online fit into this last category.

### Filter Methods

Seven different filters were applied to each of the base models to determine whether feature selection prior to modelling improved model performance.

A Univariate Cox filter fits a Cox proportional hazards model to each feature in turn. The features are ranked by the performance of the resulting Cox models, given by the C-index.

Random forest variable importance is calculated by randomly permuting a feature (i.e. adding noise to it) and calculating the difference between the prediction error before and after permutation. If the addition of noise to the feature significantly decreases the performance of the model, then that feature must be highly predictive. Features are ranked by the importance score.

Random forest minimal depth evaluates the importance of a feature by determining the shortest distance from the root of the tree to the largest subtree that has that feature as its root.^17^ The closer a feature's maximal subtree is to the root of the tree, the greater the influence it has on prediction and the more important it is. This method internally calculates a cut-off threshold.

The random forest variable hunting approach was designed for high-dimensional datasets. A forest is fitted using a randomly selected subset of features, rather than the entire set. These features are selected with probability proportional to their variable importance, with a preliminary forest run to determine the importance values. Then these features are ordered by increasing minimal depth and are added to the model one at a time until there is no increase in the joint importance. This process is repeated a specified number of times, and the variables are ranked by frequency of occurrence. A variation on this method is to order the features by their variable importance, rather than minimal depth, which is generally faster but not as accurate. This variation is not available in the software used in this analysis, so has not been included.

The maximally selected rank statistics random forest filter ranks features using the variable importance score from the maximally selected rank statistics random forest.^14^

The Minimum Redundancy Maximum Relevance filter ^18^ selects features that are mutually far away from each other, so as to minimise redundancy, while still being highly correlated with the target variable, so as to have maximum relevance.

### Wrapper Methods

Sequential forward selection, otherwise known as forward stepwise selection,^19^ starts with an empty model and at each iteration adds the feature that most improves the performance of the learning algorithm. The process is terminated when the improvement in the model performance achieved by adding the latest feature falls below a specified threshold.

Sequential forward floating selection operates in the same way except that at each iteration the algorithm evaluates the performance of all models with one additional feature and all models with one less feature.

**Supplementary Table S3: Tuning Parameters**

| **Method** | **R package** | **Function** | **Hyper-parameters and values** |
| --- | --- | --- | --- |
| **Learning Algorithms** | | | |
| Cox PH model | *survival* | coxph |  |
| Ridge | *glmnet* | cv.glmnet | alpha = 0, nfolds=5 |
| Elastic Net | *glmnet* | cv.glmnet | alpha = 0.5, nfolds=5 |
| Lasso | *glmnet* | cv.glmnet | alpha = 1, nfolds=5 |
| Likelihood based boosting with Cox models as base learners | *CoxBoost* | cv.CoxBoost |  |
| Gradient boosting with linear models as base learner | *mboost* | glmboost |  |
| Extreme Gradient Boosting – tree based | *XGBoost* | xgb.train | booster = "gbtree"  nrounds: 10 -> 25, max_depth: 1-> 10, eta: 0.01 -> 0.4 |
| Extreme Gradient Boosting – linear model based | *XGBoost* | xgb.train | booster = "gblinear".  lambda: 0 -> 50 |
| Random Survival Forests | *randomForestSRC* | rfsrc | mtry: sqrt(#features) -> 100  nodesize: 3 -> 25, ntree=1000 |
| Maximally selected rank statistics Random Forests | *ranger* | ranger | splitrule = "maxstat",  importance = "permutation",  mtry: sqrt(#features) -> 100,  min.node.size: 5 -> 50,  num.trees=1000 |
| **Feature Selection Methods (filters and wrappers)** | | | |
| Univariate Cox filter | *mlr* | various |  |
| RSF variable importance | *randomForestSRC* | rfsrc | ntree = 1000, nsplit = 10  mtry = sqrt(#features), nodesize=3 |
| RSF minimal depth | *randomForestSRC* | var.select | method = "md"  ntree=1000, nsplit=10, nodesize=3, splitrule="logrank" |
| RSF variable hunting | *randomForestSRC* | var.select | method = "vh", ntree=1000, nodesize=3, splitrule="logrank", nsplit=10, nrep=50, K=5, nstep=1 |
| MSRS RF variable importance | *ranger* | ranger | splitrule = "maxstat"  importance = "permutation"  num.trees = 1000 |
| mRMR | *mRMRe* | mRMR.classic |  |
| Sequential forward selection | *mlr* | various | alpha = 0.01, beta = -0.001 |
| Sequential forward floating selection | *mlr* | various | alpha = 0.01, beta = -0.001 |

**Supplementary Table S4: Data Preparation - MAS**

In the table below, the symbol => can be interpreted as 'combines to form'.

| Transformation | Applied to Features |
| --- | --- |
| Mean of multiple similar measurements | M_w1_systolic = AVERAGE(M_w1_sys1, M_w1_sys2, M_w1_sys3)  M_w1_diastolic = AVERAGE (M_w1_dias1, M_w1_ dias2, M_w1_ dias3)  M_w1_pulse = AVERAGE (M_w1_pulse1, M_w1_ pulse 2, M_w1_pulse 3) |
| Sum of multiple similar measurements | M_w1_VisualAcuity = SUM(M_w1_VisualAcuityR, M_w1_VisualAcuityL) |
| Largest of multiple similar measurements | M_w1_fev1 = GREATEST(M_w1_fev1_1, M_w1_fev1_2, M_w1_fev1_3)  M_w1_fvc = GREATEST(M_w1_ fvc _1, M_w1_ fvc 1_2, M_w1_ fvc 1_3)  M_w1_pef = GREATEST(M_w1_ pef _1, M_w1_ pef 1_2, M_w1_ pef 1_3)  M_w1_fer = GREATEST(M_w1_ fer _1, M_w1_ fer 1_2, M_w1_ fer 1_3) |
| Calculated a duration from a start and end date | (M_w1_smoke_start, M_w1_smoke_stop) => M_w1_years_smoked  (M_w1_motheralive, M_w1_motherdob, M_w1_motherageatdeath) => M_w1_mother_yearsalive  (M_w1_fatheralive, M_w1_fatherdob, M_w1_fatherageatdeath) => M_w1_father_yearsalive |
| Combined family history data from both parents | (M_w1_motheralz, M_w1_fatheralz) => M_w1_parentalz  (M_w1_motherparkinons, M_w1_fatherparkinsons) => M_w1_parentparkinsons |
| Combined multiple Boolean features that indicate a medical problem in similar parts of the body to form a single Boolean feature | (M_w1_asthma, M_w1_emphysema, M_w1_COPD, M_w1_chronicbronch) => M_w1_lung_probs  (M_w1_PD, M_w1_epilepsy, M_w1_brain_infection, M_w1_hydroceph, M_w1_other_brain) => M_w1_brain_probs  (M_w1_dep, M_w1_MH_other) => M_w1_mentalhealth_probs  (M_w1_vision, M_w1_glaucoma, M_w1_macdegen, M_w1_cataract, M_w1_other_eye) => M_w1_vision_probs  (M_w1_Hearing, M_w1_hearaid_recommend, M_w1_hearaid_wear) => M_w1_hearing_probs  (M_w1_ging, M_w1_perio, _w1_tooth_mob, M_w1_tooth_loss) => M_w1_dental_probs  (M_w1_snore, M_w1_sleepiness, M_w1_apnoea, M_w1_dream_act) => M_w1_sleep_probs  (M_w1_CVA, M_w1_TIA) => M_w1_stroke |
| Combined factor levels within a categorical feature to form a Boolean variable indicating a normal/abnormal condition or a yes/no or true/false response | M_w1_motora => M_w1_speech  M_w1_motorb => M_w1_face  M_w1_motorf => M_w1_STS  M_w1_motorg => M_w1_posture  M_w1_motorh => M_w1_gait  M_w1_motori => M_w1_brady  M_w1_MA1, M_w1_MA2, M_w1_MA3, M_w1_MA4, M_w1_MA5, M_w1_MA6, M_w1_MA7, M_w1_MA8, M_w1_MA9, M_w1_MA10, M_w1_MA11, M_w1_MA12, M_w1_MA13  M_w1_noticemem, M_w1_concernmem, M_w1_complainmem, M_w1_otherscomm, M_w1_drmem, M_w1_rxmem, M_w1_tiptongue, M_w1_converse, M_w1_explain, M_w1_plan, M_w1_solve, M_w1_interest_activities, M_w1_local, M_w1_unfam, M_w1_dress, M_w1_track, M_w1_appts_mem, M_w1_names |
| Combined multiple related categorical features and the factor levels within those features to form a single Boolean variable indicating a normal/abnormal condition | (M_w1_motorcface, M_w1_motorcLUL, M_w1_motorcRUL, _w1_motordL, M_w1_motordR) => M_w1_tremor  (M_w1_motoreLUL, M_w1_motoreRUL) => M_w1_rigidity |
| One-hot encoding of remaining categorical features | Sex, BORN_byRegion_MAS, hand, highqual, living, mainocc, primcare, race, balance, dentist, colour_blind, new_activities, physical_sum, confidant, regular_contact, face2face |
| Converted yes/no codings to boolean true/false | Where applicable |

In addition, the following features were omitted:

- Features with greater than 50% (MAS) or 55% (ADNI) of missing values
- Free text responses
- Features related to child or sibling data
- Features used in calculating a standardised summary score. Only the summary score itself was included in the model.
- Conditional responses. In some cases, additional information was provided if an initial condition was met. For example, if a patient had ever suffered a stroke, then they were asked for additional related information such as the number of strokes they had suffered. That additional information was omitted, as it was not applicable in the majority of cases.

**Supplementary Table S5: Data Cleaning and Preparation – ADNI**

| Transformation | Applied to Features |
| --- | --- |
| Convert yes/no to boolean | Where applicable |
| Converted strings to factors | NBAT.LMSTORY |
| Converted values to consistent scales in vitals | VSHEIGHT -> centimeters  VSTEMP -> celsius  VSWEIGHT -> kilograms |
| Extracted numerical information from strings | UPENNBIOMK.COMMENT  MODHACH  NPIQ SEV |

In addition, the following features were omitted:

- Features with greater than 55% of missing data
- Free text responses
- Features related to child or sibling data
- Features used in calculating a standardised summary score. Only the summary score itself was included in the model.
- Conditional responses. In some cases, additional information was provided if an initial condition was met. For example, if a patient had ever suffered a stroke, then they were asked for additional related information such as the number of strokes they had suffered. That additional information was omitted, as it was not applicable in the majority of cases.

**Supplementary Table S6: Full Results**

Results show average value of the Concordance Index over 5 repeats of 5-fold cross validation. Figures in brackets show standard deviation

*Indicates that the performance of the method was statistically significantly worse than the best-performing model (in bold), p-value = 0.05.

| **Model/Filter** | **Dataset** | **(1)  None** | **(2) Univariate** | **(3)  RF Var Imp** | **(4)  RF Min Depth** | **(6)  RF Max Stat** | **(7)  mRMR** | **(8)  SFS** | **(9)  SFFS** |
| --- | --- | --- | --- | --- | --- | --- | --- | --- | --- |
| **CoxPH (1)** | **MAS** | 0.61 (0.06) * | 0.81 (0.06) | 0.82 (0.05) | 0.82 (0.06) | 0.80 (0.06) | 0.51 (0.06)* | 0.79 (0.05) | 0.78 (0.06) |
|  | **ADNI** | 0.86 (0.15) | 0.93 (0.02) | 0.92 (0.02) | 0.92 (0.02) | 0.93 (0.02) | 0.50 (0.00)* | 0.91 (0.02) | 0.92 (0.02) |
| **Ridge (2)** | **MAS** | 0.80 (0.06) | 0.81 (0.06) | 0.81 (0.06) | 0.81 (0.05) | 0.81 (0.05) | 0.57 (0.00)* |  |  |
|  | **ADNI** | 0.92 (0.02)* | 0.93 (0.01) | 0.92 (0.02) | **0.93 (0.01)** | 0.93 (0.01) |  |  |  |
| **ElasticNet (3)** | **MAS** | 0.81 (0.05) | 0.82 (0.05) | 0.82 (0.05) | 0.82 (0.05) | 0.82 (0.05) | 0.50 (0.00)* |  |  |
|  | **ADNI** | 0.93 (0.01) | 0.93 (0.02) | 0.93 (0.01) | 0.93 (0.01) | 0.93 (0.01) |  |  |  |
| **Lasso (4)** | **MAS** | 0.81 (0.05) | 0.81 (0.05) | 0.82 (0.05) | 0.82 (0.05) | 0.82 (0.05) | 0.50 (0.08)* |  |  |
|  | **ADNI** | 0.93 (0.02) | 0.93 (0.02) | 0.93 (0.01) | 0.93 (0.01) | 0.93 (0.01) |  |  |  |
| **CoxBoost (5)** | **MAS** | 0.82 (0.05) | 0.82 (0.05) | 0.82 (0.05) | **0.83 (0.06)** | 0.82 (0.05) | 0.55 (0.07)* |  |  |
|  | **ADNI** | 0.93 (0.01) | 0.93 (0.02) | 0.93 (0.02) | 0.93 (0.02) | 0.93 (0.02) | 0.50 (0.00)* |  |  |
| **GLMBoost (6)** | **MAS** | 0.79 (0.05) | 0.79 (0.06) | 0.79 (0.05) | 0.79 (0.05) | 0.79 (0.05) | 0.56 (0.07)* | 0.76 (0.04)* | 0.77 (0.05)* |
|  | **ADNI** | 0.93 (0.02) | 0.93 (0.02) | 0.93 (0.02) | 0.93 (0.02) | 0.93 (0.01) | 0.50 (0.00)* | 0.91 (0.02)* | 0.92 (0.02) |
| **XGBoost - Tree (7)** | **MAS** | 0.79 (0.06) | 0.69 (0.07)* | 0.71 (0.06)* | 0.72 (0.07)* | 0.71 (0.06)* | 0.51 (0.05)* | 0.70 (0.06)* | 0.69 (0.05)* |
|  | **ADNI** | 0.93 (0.02) | 0.91 (0.02)* | 0.91 (0.02)* | 0.91 (0.02)* | 0.91 (0.02)* | 0.50 (0.00)* | 0.90 (0.02)* | 0.91 (0.02)* |
| **XGBoost - Lin (8)** | **MAS** | 0.80 (0.05) | 0.81 (0.05) | 0.80 (0.06) | 0.81 (0.06) | 0.80 (0.06) | 0.58 (0.08)* | 0.78 (0.05) | 0.79 (0.05) |
|  | **ADNI** | 0.91 (0.02)* | 0.91 (0.01)* | 0.91 (0.02) | 0.92 (0.02)* | 0.91 (0.02)* | 0.50 (0.00)* | 0.91 (0.02)* | 0.93 (0.02)* |
| **RF-Survival (9)** | **MAS** | 0.80 (0.06) | 0.79 (0.06)* | 0.80 (0.06) | 0.79 (0.06) | 0.80 (0.06) | 0.58 (0.08)* | 0.71 (0.08)* | 0.71 (0.05)* |
|  | **ADNI** | 0.92 (0.01) | 0.87 (0.02)* | 0.88 (0.02)* | 0.88 (0.02)* | 0.87 (0.02)* | 0.51 (0.18)* | 0.86 (0.03)* | 0.87 (0.02)* |
| **RF-MaxStat (10)** | **MAS** | 0.81 (0.05) | 0.81 (0.06) | 0.81 (0.06) | 0.81 (0.06) | 0.81 (0.06) | 0.59 (0.07)* | 0.74 (0.05)* | 0.74 (0.05)* |
|  | **ADNI** | 0.90 (0.02)* | 0.88 (0.02)* | 0.88 (0.02)* | 0.88 (0.02)* | 0.88 (0.02)* | 0.50 (0.00)* | 0.89 (0.02)* | 0.89 (0.02)* |


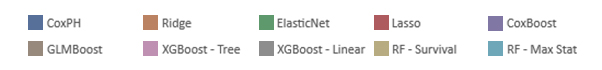

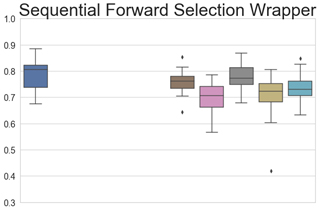

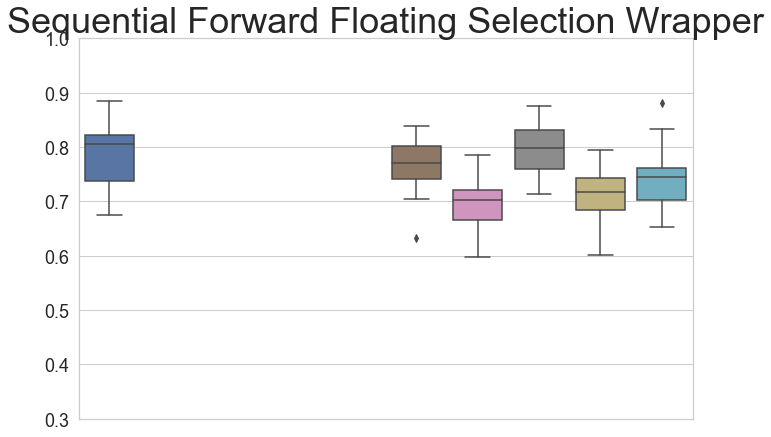

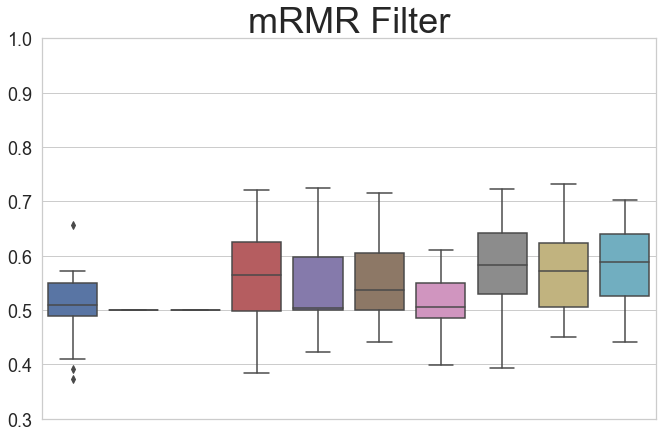

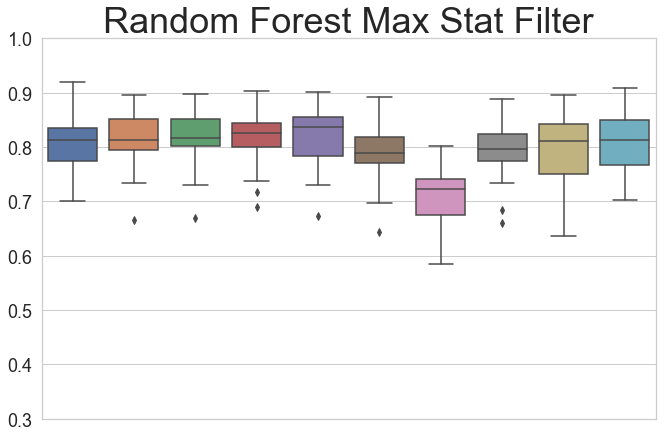

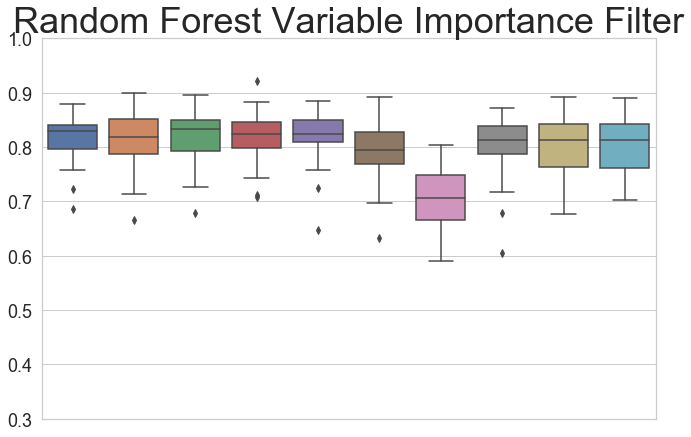

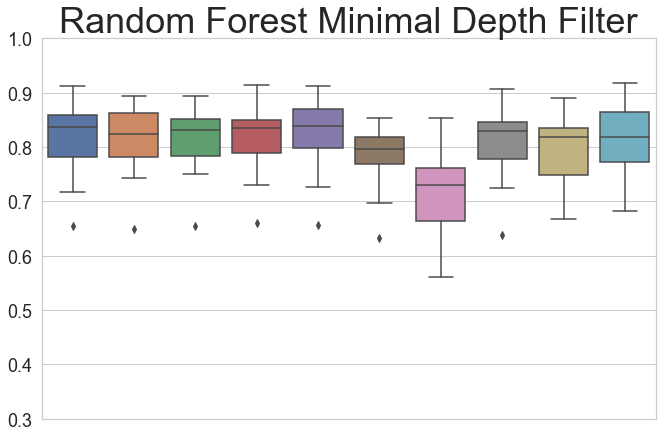

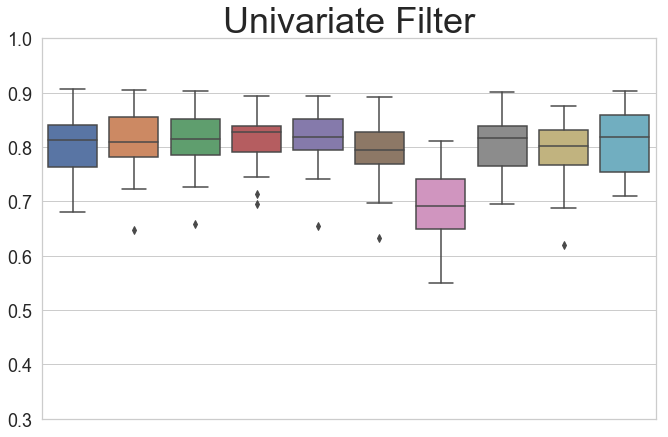

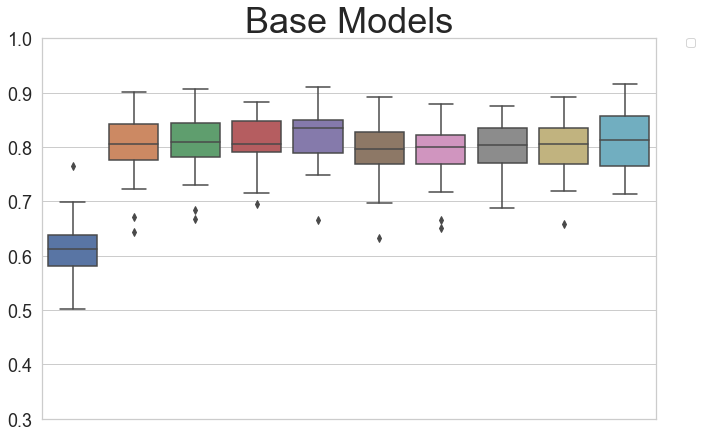


**Supplementary Figure S7 - *Boxplots showing the spread of values of the C-Index produced by each of the models - MAS***


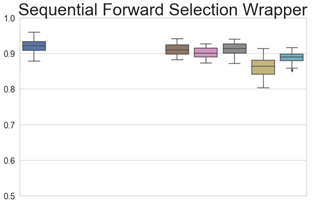

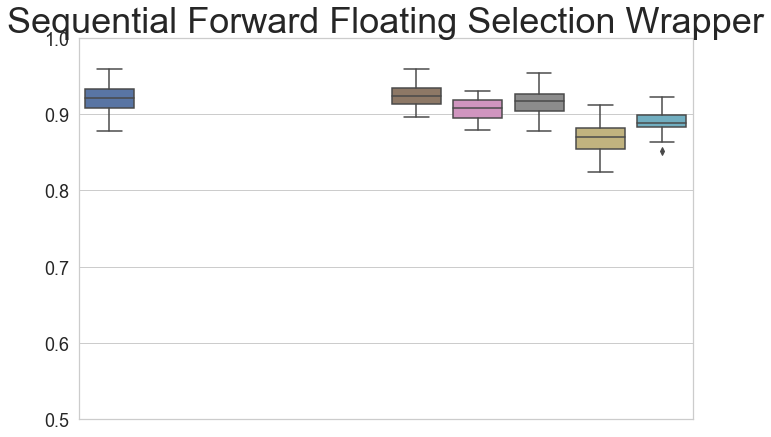

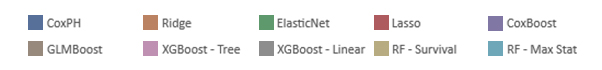

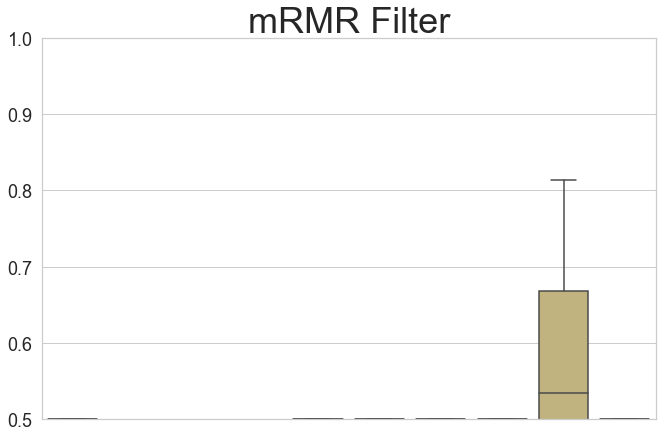

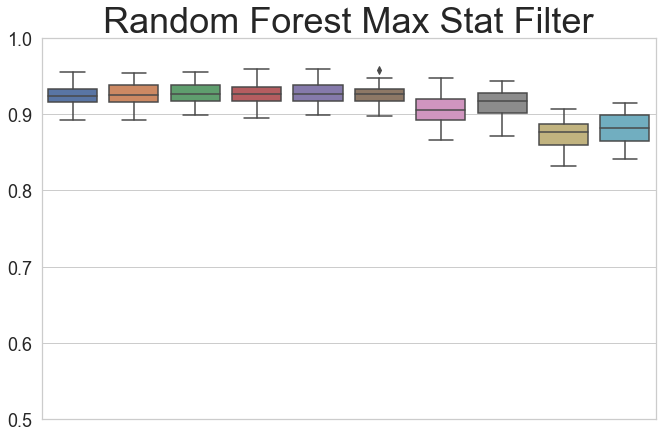

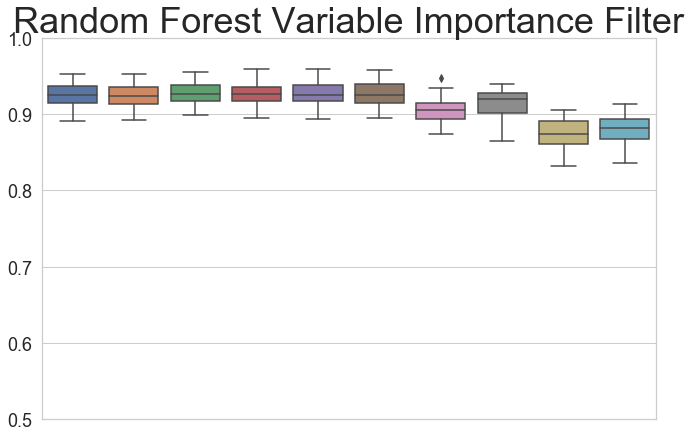

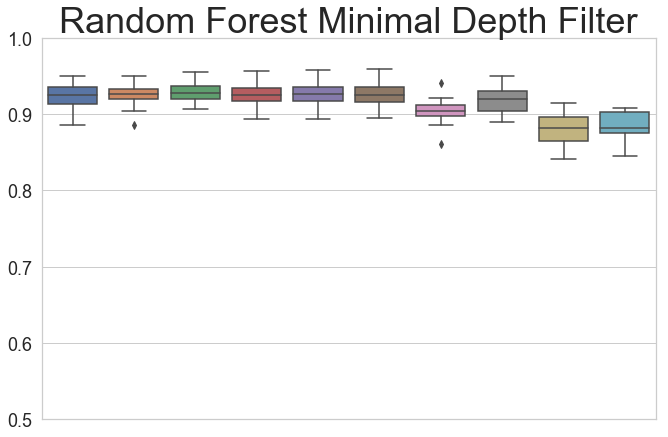

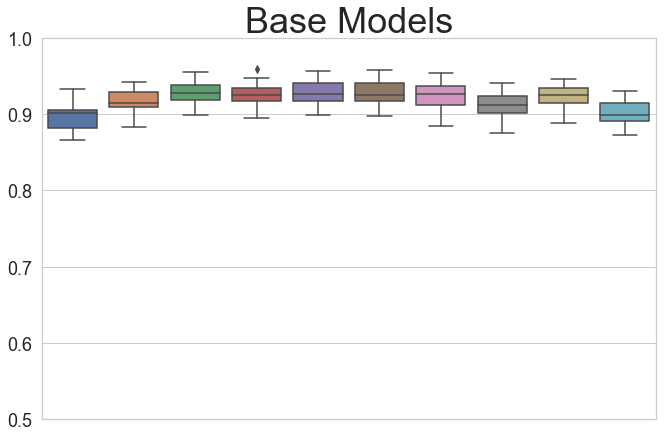

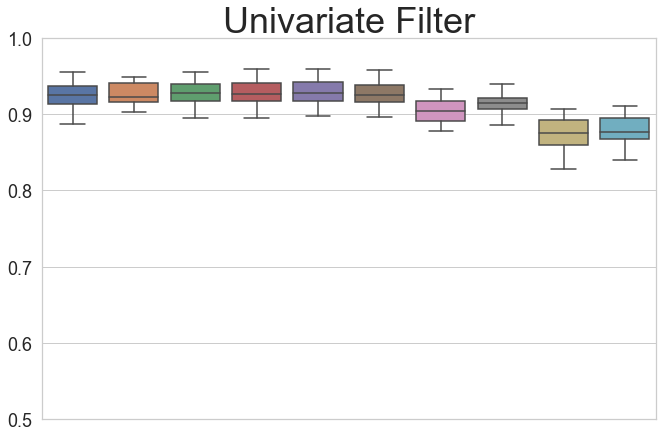
**Supplementary Figure S8 - *Boxplots showing the spread of values of the C-Index produced by each of the models - ADNI***

# References

1. Cox, D. R. Regression Models and Life-Tables. *J. R. Stat. Soc.* **34**, 187–220 (1972).

2. Zou, H. & Hastie, T. Regularization and variable selection via the elastic net. *J. R. Stat. Soc. Ser. B Stat. Methodol.* **67**, 301–320 (2005).

3. Tibshirani, R. J. The lasso method for variable selection in the Cox model. *Stat. Med.* **16**, 385–395 (1997).

4. Simon, N., Friedman, J., Hastie, T. & Tibrishani, R. Regularization Paths for Cox’s Proportional Hazards Model via Coordinate Descent. 1–13 (2011). doi:10.1038/nm.2451.A

5. Schapire, R. E. The Strength of Weak Learnability (Extended Abstract). *Mach. Learn.* **5**, 197–227 (1990).

6. Friedman, J., Hastie, T. & Tibshirani, R. Additive logistic regression: a statistical view of boosting (With discussion and a rejoinder by the authors). *Ann. Stat.* **28**, 337–407 (2000).

7. Hothorn, T., Bühlmann, P., Dudoit, S., Molinaro, A. & Van Der Laan, M. J. Survival ensembles. *Biostatistics* **7**, 355–373 (2006).

8. Tutz, G. & Binder, H. Generalized additive modeling with implicit variable selection by likelihood-based boosting. *Biometrics* **62**, 961–971 (2006).

9. Friedman, J. H. Stochastic gradient boosting. *Comput. Stat. Data Anal.* **38**, 367–378 (2002).

10. Chen, T. & Guestrin, C. XGBoost: A Scalable Tree Boosting System Tianqi. *Proc. 22nd ACM SIGKDD Int. Conf. Knowl. Discov. Data Min.* 785–794 (2016).

11. Ishwaran, H., Kogalur, U. B., Blackstone, E. H. & Lauer, M. S. Random survival forests. *Ann. Appl. Stat.* **2**, 841–860 (2008).

12. Breiman, L. Random Forests. *Mach. Learn.* 1–33 (2001). doi:10.1017/CBO9781107415324.004

13. Hothorn, T., Hornik, K. & Zeileis, A. Unbiased recursive partitioning: A conditional inference framework. *J. Comput. Graph. Stat.* **15**, 651–674 (2006).

14. Wright, M. N., Dankowski, T. & Ziegler, A. Unbiased split variable selection for random survival forests using maximally selected rank statistics. *Stat. Med.* **36**, 1272–1284 (2017).

15. Lausen, B. & Schumacher, M. Maximally Selected Rank Statistics. *Biometrics* **48**, 73–85 (1992).

16. Guyon, I., Elisseeff, A. & De, A. M. An Introduction to Variable and Feature Selection. *J. Mach. Learn. Res.* **3**, 1157–1182 (2003).

17. Ishwaran, H., Kogalur, U. B., Gorodeski, E. Z., Minn, A. J. & Lauer, M. S. High-dimensional variable selection for survival data. *J. Am. Stat. Assoc.* **105**, 205–217 (2010).

18. Peng, H., Long, F. & Ding, C. Feature Selection Based on Mutual Information: Criteria of Max-Dependency, Max-Relevance, and Min-Redundancy. *IEEE Trans Pattern Anal Mach Intell* **27**, 1226–1238 (2005).

19. Hastie, T., Tibrishani, R. & Friedman, J. *The Elements of Statistical Learning: Data Mining, Inference, and Prediction*. (Springer Series in Statistics, 2017).
